# COVID-19 trends and severity among symptomatic children aged 0–17 years in 10 European Union countries, 3 August 2020 to 3 October 2021

**DOI:** 10.2807/1560-7917.ES.2021.26.50.2101098

**Published:** 2021-12-16

**Authors:** Nick Bundle, Nishi Dave, Anastasia Pharris, Gianfranco Spiteri, Charlotte Deogan, Jonathan E Suk, Study group members, Study group members, Soteroulla Soteriou, Anna Papandreou, Valentinos Silvestros, Maria Athanasiadou, Theopisti Kyprianou, Anna Demetriou, Otto Helve, Emmi Sarvikivi, Silke Buda, Barbara Hauer, Walter Haas, Thorsten Wolff, Aoife Colgan, Kate O’Donnell, Antonino Bella, Joël Mossong, Anne Vergison

**Affiliations:** 1 European Centre for Disease Prevention and Control (ECDC), Stockholm, Sweden; 2 The Study group members are listed under Investigators

**Keywords:** COVID-19, surveillance, Europe, children

## Abstract

We estimated risks of severe outcomes in 820,404 symptomatic paediatric COVID-19 cases reported by 10 European Union countries between August 2020 and October 2021. Case and hospitalisation rates rose as transmission increased but severe outcomes were rare: 9,611 (1.2%) were hospitalised, 640 (0.08%) required intensive care and 84 (0.01%) died. Despite increased individual risk (adjusted odds ratio hospitalisation: 7.3; 95% confidence interval: 3.3–16.2; intensive care: 8.7; 6.2–12.3) in cases with comorbidities, most (83.7%) hospitalised children had no comorbidity.

Understanding the burden of coronavirus disease (COVID-19) among children is essential for evidence-based decision-making regarding the vaccination of children and for assessing the importance of severe acute respiratory syndrome coronavirus 2 (SARS-CoV-2) mitigation measures in specific settings, such as schools [1]. Here, we report on the burden and severity of symptomatic notified COVID-19 cases among children in the European Union (EU).

## Understanding the burden of COVID-19 in children in Europe

We analysed, using R v4.1.1 (R Core Team, Vienna, Austria [2]), pooled case-based surveillance data reported to The European Surveillance System (TESSy) by 10 EU countries (Austria, Cyprus, Finland, Germany, Ireland, Italy, Luxembourg, Malta, Slovakia and Sweden) for symptomatic COVID-19 cases (reported as symptomatic or with a date of onset), between weeks 32/2020 and 43/2021. We restricted the analysis to weeks 32/2020 to 39/2021 to account for delayed reporting of the outcomes: hospitalisation, intensive care unit (ICU) (admission to ICU and/or requiring ventilation or extracorporeal membrane oxygenation (ECMO)) or death. We compared the cumulative number of reported deaths by each country to official data from public sources [3], with a minimum completeness threshold of 90% for inclusion in this study. Time series of reported hospital and ICU admissions were compared with those for admission or occupancy obtained from public sources [4], excluding countries reporting incomplete time series or with peaks that occurred at different times. Outcomes reported as ‘unknown’ were recoded to ‘no’ for deaths in all countries, and for hospitalisation/ICU in Ireland (following national practice) and Sweden (all cases were coded as 'yes’ or ‘unknown’). Hospitalisation/ICU status was recoded to ‘no’ if date of hospitalisation preceded date of onset, to minimise inclusion of incidental hospital admissions in the crude risk numerators. Cases with unknown outcome or sex were excluded.

We described trends by age group in weekly rates and proportions of case notifications and hospital admissions. Age-specific cumulative case notification rates per 100,000 population and crude risk (percentage of cases in each age group reporting the outcome) for hospitalisation, ICU admission (among all and hospitalised cases) and death were estimated by age group in years and in months < 2 years using data from seven countries that reported age in months (Austria, Cyprus, Finland, Ireland, Luxembourg, Slovakia and Sweden). We considered the overall study period and the period when the SARS-CoV-2 Delta variant of concern (Phylogenetic Assignment of Named Global Outbreak (Pango) lineage designation B.1.617.2) accounted for more than 95% of reported weekly sequences (median: weeks 28/2021 to 39/2021). Age-specific distributions of the outcomes by period and sex were compared using chi-squared tests.

Cases with unknown comorbidity were excluded from a subset of data from seven countries (Cyprus, Finland, Italy, Luxembourg, Malta, Slovakia and Sweden) reporting data on comorbidities (coded as cancer, diabetes, cardiac disease, lung disease, neuromuscular disease, HIV infection, asthma, kidney disease, hypertension, pregnancy, liver disease, obesity, smoker/history of smoking). We estimated crude risks stratified by the presence and absence of a comorbidity and adjusted odds ratios (aOR; via logistic regression) to compare outcomes for cases in each age group (< 1, 1–4, 5–11, 12–17, 0–17 years) with and without any comorbidity.

## Trends in case notification and hospitalisation

**Figure 1:**
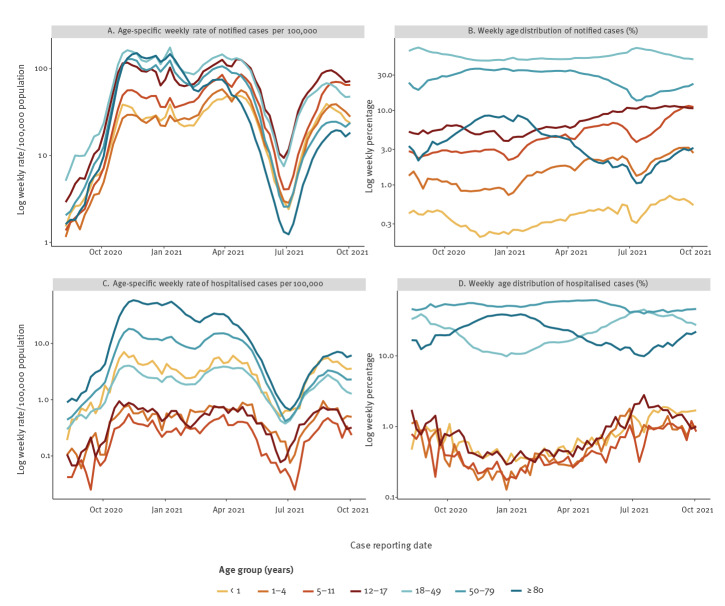
Weekly age distribution and rates of notified symptomatic COVID-19 cases and hospital admissions reported to TESSy, pooled from 10 EU countries, weeks 32/2020 to 39/2021 (n = 6,604,483)

COVID-19: coronavirus disease; EU: European Union; TESSy: The European Surveillance System.

Pooled weekly notification rates increased sharply in all age groups from July 2021 (Figure 1A). We observed concomitant rises in hospitalisation rates in all age groups, but starting from, and reaching, much lower levels in children aged 1–17 years than in adults or children younger than 1 year (Figure 1C). Since January 2021, children have represented an increasing proportion of notified cases and hospital admissions (Figure 1B and D).

Cumulative case rates reported among a total of 820,404 symptomatic paediatric cases (12.4% of 6,604,483 cases of all ages) increased with every additional year of age from 2 to 17 years (Table 1). Hospitalisation was reported for 9,611 (1.2%) cases, ICU admission for 640 (0.08% of all cases, 6.7% of hospitalised cases) and death for 84 (0.01%). Limiting the analysis to children aged 2–17 years, the overall risks of these outcomes were 0.8%, 0.06%, 7.5% and 0.01%, respectively. The risk of hospitalisation was highest among the youngest (0–2 months), with point estimates that decreased with increasing age to 9 years and then increased with each year from 12 to 17 years. Male children aged 0–17 years were slightly more likely than female children to be admitted to hospital (1.2% vs 1.1%; p < 0.0001) or ICU (0.09% vs 0.07%; p < 0.05), but these differences did not exist in all paediatric age groups. Results for periods coinciding with dominance of the Delta variant were consistent with the full study period, although hospitalisation was more common among children younger than 1 year (14.7% Delta vs 13.1% full period; p < 0.01).

**Table 1:** Age-specific counts and crude risks of severe COVID-19 outcomes by age, pooled from 10 EU countries, weeks 32/2020 to 39/2021 (n = 6,604,483)

Age	Symptomatic cases	Cases per 100,000 population	Hospitalisation		ICU		Death		ICU among hospitalised cases
			n	Crude risk % (95% CI)	n	Crude risk % (95% CI)	n	Crude risk % (95% CI)	Crude risk % (95% CI)
Total 0–23 months^a^	8,258	NS	695	8.42 (7.83–9.04)	95	1.15 (0.93–1.40)	8	0.10 (0.04–0.19)	13.67 (11.20–16.45)
0–2 months	1,243	NS	320	25.74 (23.33–28.27)	39	3.14 (2.24–4.26)	3	0.24 (0.05–0.70)	12.19 (8.81–16.28)
3–5 months	869	NS	101	11.62 (9.57–13.94)	18	2.07 (1.23–3.25)	0	0	17.82 (10.92–26.70)
6–8 months	995	NS	64	6.43 (4.99–8.14)	11	1.11 (0.55–1.97)	2	0.20 (0.02–0.72)	17.19 (8.90–28.68)
9–11 months	1,085	NS	63	5.81 (4.49–7.37)	6	0.55 (0.20–1.20)	0	0	9.52 (3.58–19.59)
12–17 months	2,042	NS	82	4.02 (3.21–4.96)	7	0.34 (0.14–0.71)	2	0.10 (0.01–0.35)	8.54 (3.50 - 16.80)
18–23 months	2,024	NS	65	3.21 (2.49–4.08)	14	0.69 (0.38–1.16)	1	0.05 (0.00–0.27)	21.54 12.31–33.49)
Total 0–17 years	820,404	2,692	9,611	1.17 (1.15–1.20)	640	0.08 (0.07–0.08)	84	0.01 (0.01–0.01)	6.66 (6.17–7.18)
< 1 year	22,518	1,432	2,952	13.11 (12.67–13.56)	142	0.63 (0.53–0.74)	15	0.07 (0.04–0.11)	4.81 (4.07–5.65)
1 year	24,797	1,525	852	3.44 (3.21–3.67)	44	0.18 (0.13–0.24)	5	0.02 (0.01–0.05)	5.16 (3.78–6.87)
2 years	23,032	1,384	471	2.04 (1.87–2.24)	22	0.10 (0.06–0.14)	4	0.02 (0.00–0.04)	4.67 (2.95–6.99)
3 years	24,573	1,452	324	1.32 (1.18–1.47)	14	0.06 (0.03–0.10)	3	0.01 (0.00–0.04)	4.32 (2.38–7.14)
4 years	24,858	1,484	265	1.07 (0.94–1.20)	15	0.06 (0.03–0.10)	6	0.02 (0.01–0.05)	5.66 (3.20–9.16)
5 years	26,494	1,574	241	0.91 (0.80–1.03)	18	0.07 (0.04–0.11)	4	0.02 (0.00–0.04)	7.47 (4.49–11.55)
6 years	31,300	1,881	225	0.72 (0.63–0.82)	20	0.06 (0.04–0.10)	1	0.00 (0.00–0.02)	8.89 (5.51–13.39)
7 years	34,857	2,062	230	0.66 (0.58–0.75)	14	0.04 (0.02–0.07)	3	0.01 (0.00–0.03)	6.09 (3.37–10.00)
8 years	39,241	2,325	253	0.64 (0.57–0.73)	23	0.06 (0.04–0.09)	3	0.01 (0.00–0.02)	9.09 (5.85–13.33)
9 years	44,703	2,588	266	0.60 (0.53–0.67)	24	0.05 (0.03–0.08)	0	0	9.02 (5.87–13.13)
10 years	50,126	2,914	315	0.63 (0.56–0.70)	31	0.06 (0.04–0.09)	3	0.01 (0.00–0.02)	9.84 (6.79–13.68)
11 years	54,337	3,120	307	0.56 (0.50–0.63)	29	0.05 (0.04–0.08)	4	0.01 (0.0–0.02)	9.45 (6.42–13.28)
12 years	58,210	3,371	328	0.56 (0.50–0.63)	26	0.04 (0.03–0.07)	4	0.01 (0.00–0.02)	7.93 (5.24–11.40)
13 years	61,897	3,621	374	0.60 (0.54–0.67)	35	0.06 (0.04–0.08)	6	0.01 (0.00–0.02)	9.36 (6.61–12.77)
14 years	63,855	3,737	430	0.67 (0.61–0.74)	49	0.08 (0.06–0.10)	8	0.01 (0.01–0.02)	11.40 (8.55–14.78)
15 years	70,862	4,091	477	0.67 (0.61–0.74)	38	0.05 (0.04–0.07)	7	0.01 (0.00–0.02)	7.97 (5.70–10.77)
16 years	77,353	4,490	567	0.73 (0.67–0.80)	40	0.05 (0.04–0.07)	1	0.00 (0.00–0.01)	7.05 (5.09–9.48)
17 years	87,391	5,043	734	0.84 (0.78–0.90)	56	0.06 (0.05–0.08)	7	0.01 (0.00–0.02)	7.63 (5.81–9.79)
1–4 years	97,260	1,461	1,912	1.97 (1.88–2.06)	95	0.10 (0.08–0.12)	18	0.02 (0.01–0.03)	4.97 (4.04–6.04)
5–11 years	281,058	2,359	1,837	0.65 (0.62–0.68)	159	0.06 (0.05–0.07)	18	0.01 (0.00–0.01)	8.66 (7.41–10.04)
12–17 years	419,568	4,061	2,910	0.69 (0.67–0.72)	244	0.06 (0.05–0.07)	33	0.01 (0.01–0.01)	8.38 (7.40–9.45)
18–49 years	3,339,485	4,702	86,961	2.60 (2.59–2.62)	10,293	0.31 (0.30–0.31)	2,837	0.08 (0.08–0.09)	11.84 (11.62–12.05)
50–79 years	2,095,032	3,143	277,377	13.24 (13.19–13.29)	59,911	2.86 (2.84–2.88)	73,672	3.52 (3.49–3.54)	21.60 (21.45–21.75)
≥ 80 years	349,562	2,948	141,502	40.48 (40.32–40.64)	16,136	4.62 (4.55–4.69)	116,871	33.43 (33.28–33.59)	11.40 (11.24–11.57)

CI: confidence interval; COVID-19: coronavirus disease; EU: European Union; ICU: intensive care unit; NS: not shown.

^a^Analysis of a subset of cases younger than 2 years with data on age in months, from seven countries (Austria, Cyprus, Finland, Ireland, Luxembourg, Slovakia and Sweden). Case rates per 100,000 population are not provided for these age groups.

## Stratification by presence of any comorbidity

Data on comorbidities were available for 210,008 of 460,790 paediatric cases (45.6%) from the seven countries that provided this information. Among these, 203,548 (96.9%) reported having none, 5,773 (2.7%) one and 687 (0.3%) two or more comorbidities. Data on comorbidities were less likely to be reported for hospitalised than non-hospitalised paediatric cases (30.0% vs 45.8%; p < 0.0001). After controlling for age group, reporting country, sex and four periods (weeks 32/2020 to 53/2020 (pre-vaccine period), 1/2021 to 21/2021 (Alpha (B.1.1.7) variant dominant), 22/2021 to 27/2021 (Alpha and Delta variants co-circulating), 28/2021 to 39/2021 (Delta variant dominant)), the adjusted odds of hospitalisation, ICU admission and death were seven, nine and 27 times higher, respectively, among cases with at least one comorbidity compared with those with none (Table 2).

**Table 2:** Age-specific counts, crude risks and adjusted odds ratios for severe COVID-19 outcomes comparing cases with and without any comorbidity, pooled from seven EU countries, weeks 32/2020 to 39/2021 (n = 210,008)

Age (years)	Any comorbidity	Symptomatic cases	Cases with outcome	Crude stratified risk		Adjusted RR^a^
				Risk % (95% CI)	p value	aOR (95% CI)
Hospitalisation						
Total 0–17	No	203,548	1,506	0.74 (0.70–0.78)	< 0.0001	7.29 (3.28–16.20)
	Yes	6,460	293	4.54 (4.04–5.07)		
< 1	No	3,812	537	14.09 (13.00–15.23)	< 0.05	1.68 (0.38–7.41)
	Yes	184	37	20.11 (14.57–26.64)		
1–4	No	16,054	275	1.71 (1.52–1.93)	< 0.0001	8.95 (5.02–15.95)
	Yes	419	56	13.37 (10.26–17.00)		
5–11	No	68,152	273	0.40 (0.35–0.45)	< 0.0001	11.98 (9.02–15.91)
	Yes	1,791	77	4.30 (3.41–5.34)		
12–17	No	115,530	421	0.36 (0.33–0.40)	< 0.0001	9.28 (5.92–14.54)
	Yes	4,066	123	3.03 (2.52–3.60)		
ICU						
Total 0–17	No	203,548	102	0.05 (0.04–0.06)	< 0.0001	8.74 (6.22–12.27)
	Yes	6,460	32	0.50 (0.34–0.70)		
< 1	No	3,812	32	0.84 (0.57–1.18)	1	1.72 (0.30–9.96)
	Yes	184	2	1.09 (0.13–3.87)		
1–4	No	16,054	21	0.13 (0.08–0.20)	< 0.0001	10.25 (5.70–18.45)
	Yes	419	6	1.43 (0.53–3.09)		
5–11	No	68,152	15	0.02 (0.01–0.04)	< 0.0001	18.56 (10.12–34.06)
	Yes	1,791	9	0.50 (0.23–0.95)		
12–17	No	115,530	34	0.03 (0.02–0.04)	< 0.0001	10.25 (7.55–13.90)
	Yes	4,066	15	0.37 (0.21–0.61)		
Death						
Total 0–17	No	203,548	22	0.01 (0.01–0.02)	< 0.0001	26.85 (4.97–145.05)
	Yes	6,460	14	0.22 (0.12–0.36)		
< 1	No	3,812	4	0.10 (0.03–0.27)	0.56	9.05 (0.70–117.78)
	Yes	184	1	0.54 (0.01–2.99)		
1–4	No	16,054	6	0.04 (0.01–0.08)	0.44	7.02 (0.62–79.09)
	Yes	419	1	0.24 (0.01–1.32)		
5–11	No	68,152	2	0.00 (0.00–0.01)	< 0.0001	158.61 (19.06–1,319.58)
	Yes	1,791	6	0.34 (0.12–0.73)		
12–17	No	115,530	10	0.01 (0.00–0.02)	< 0.0001	24.81 (4.49–137.05)
	Yes	4,066	6	0.15 (0.05–0.32)		
ICU among hospitalised cases						
Total 0–17	No	1,506	102	6.77 (5.56–8.16)	< 0.05	1.24 (0.95–1.62)
	Yes	293	32	10.92 (7.59–15.07)		
< 1	No	537	32	5.96 (4.11–8.31)	1	0.73 (0.29–1.88)
	Yes	37	2	5.41 (0.66–18.19)		
1–4	No	275	21	7.64 (4.79–11.44)	0.62	1.37 (0.65–2.89)
	Yes	56	6	10.71 (4.03–21.88)		
5–11	No	273	15	5.49 (3.11–8.90)	0.1	1.58 (0.94–2.66)
	Yes	77	9	11.69 (5.49–21.03)		
12–17	No	421	34	8.08 (5.66–11.10)	0.22	1.18 (0.74–1.90)
	Yes	123	15	12.20 (6.99–19.32)		

COVID-19: coronavirus disease; EU: European Union; ICU: intensive care unit; RR: relative risk.

^a^Logistic regression models: for Total 0–17 years: outcome ca any comorbidity + age group (< 1, 1–4, 5–11, 12–17 years) + covariables period (weeks 32/2020 to 53/2020 (pre-vaccine period), 1/2021 to 21/2021 (Alpha variant dominant), 22/2021 to 27/2021 (Alpha and Delta variants co-circulating), 28/2021 to 39/2021 (Delta variant dominant), sex (male/female) and reporting country. Robust standard errors accounting for clustering on reporting country. Age group-specific estimates were obtained using an interaction between age group and any comorbidity.

This table is based on data from seven countries (Cyprus, Finland, Italy, Luxembourg, Malta, Slovakia and Sweden) with information on comorbidities reported for 210,008 of 460,790 paediatric cases (45.6%). Data from Austria, Germany and Ireland (359,614 cases, 3,619 hospitalised) that did not include information on comorbidities were excluded.

## Distribution of comorbidities among hospitalised cases

**Figure 2:**
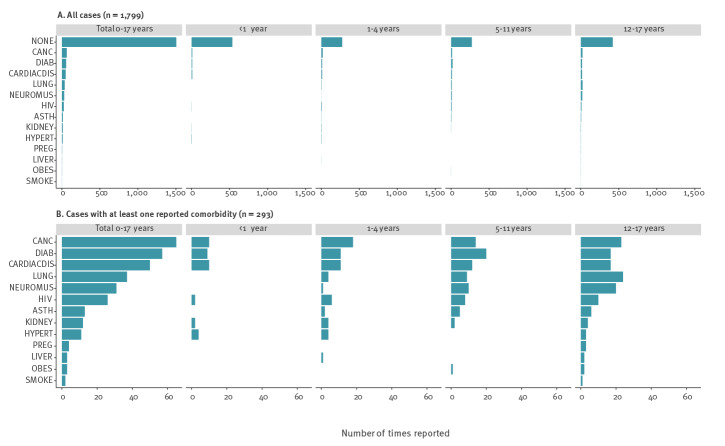
Occurrence of reported comorbidities among hospitalised paediatric COVID-19 cases by age group, pooled from seven EU countries, weeks 32/2020 to 39/2021 (n = 1,799)

Of the 210,008 paediatric cases with data on comorbidities, 83.7% (1,506/1,799) of those hospitalised were reported not to have any. This proportion fell with increasing age: 93.6% (37/574; < 1 year), 83.1% (56/331; 1–4 years), 78.0% (77/350; 5–11 years) and 77.4% (123/544; 12–17 years) (Figure 2A). Among 293 (16.3%) hospitalised cases reporting a total of 314 comorbidities (18 cases had two and one case had four comorbidities), the most frequently reported conditions were cancer (20.7%), diabetes (18.2%), cardiac (15.9%) and lung (11.8%) disease (Figure 2B). Cancer (n = 7) followed by HIV (n = 5), lung disease (n = 5), cardiac disease (n = 4) and diabetes (n = 4) were most common among the 34 of 136 (25.0%) cases admitted to ICU reporting at least one comorbidity, and 14 of 36 fatal cases had at least one comorbidity.

ASTH: asthma; CANC: cancer; CARDIACDIS: cardiac disease; COVID-19: coronavirus disease; DIAB: diabetes; EU: European Union; HIV: human immunodeficiency virus; HYPERT: hypertension; KIDNEY: kidney disease; LIVER: liver disease; LUNG: lung disease; NEUROMUS: neuromuscular disease; OBES: obesity; PREG: pregnancy (including post-partum); SMOKE: smoker/history of smoking.

One instance each of smoker and pregnancy reported in two infants aged 0 months (< 1 year) are not shown in the figure.

## Discussion

By week 47/2021 only 15.2% (range: 1.0–29.0%) of children younger than 18 years in the EU and European Economic Area (EEA) had been fully vaccinated against COVID-19 [5]. The European Medicines Agency (EMA) recently approved the emergency authorisation of Comirnaty vaccine (BNT162b2 mRNA, BioNTech-Pfizer, Mainz, Germany/New York, United States (US)) in children aged 5–11 years [6]. The evidence presented here indicates that case notification and hospital admission rates among children rise as overall transmission increases, but that most children with symptomatic COVID-19 have a very low risk of death or hospitalisation. For every 10,000 symptomatic paediatric cases reported during the study period, ca 117 were hospitalised and eight required ICU admission or respiratory support.

The high COVID-19 incidence in many parts of the EU/EEA means that large numbers of unvaccinated children are likely to be exposed to the virus, leading to increases in the absolute numbers of children with severe COVID-19 outcomes. In addition, perhaps particularly in countries that have achieved high levels of vaccination coverage in adults, the majority of community transmission could be increasingly among children [7]. Thus, tailored national guidance and appropriate mitigation measures, notably in schools and other places where children congregate, will continue to be essential [1].

This study highlights that the risk of a severe COVID-19 outcome is substantially elevated for children with underlying risk factors compared with healthy children [8]. However, among paediatric COVID-19 cases with information on comorbidities, 83.7% had no reported comorbidity, demonstrating a potential population-level impact of high levels of community transmission leading to large numbers of hospital admissions among healthy children. 

The elevated risk of hospitalisation observed for children younger than 2 years may reflect lower thresholds for admission of infants and neonates in particular [9], and further research is required to clarify the risk of severe disease in this age group. Whether the higher crude risk of hospitalisation among children under 1 year in the period of dominance of the SARS-CoV-2 Delta variant reflects elevated severity of this variant is unclear since this was not observed in other age groups.

This analysis did not consider other health impacts of COVID-19 on children such as the prevalence and burden of paediatric inflammatory multisystem syndrome or post-COVID-19 syndrome, as well as the numerous indirect negative health and mental health impacts on children caused by disruptions to their social and educational lives [1]. Such factors are, nonetheless, important considerations for decision making about vaccination of children [10].

There are important limitations to this study. The analysis is based on surveillance data reported to TESSy with at least 90% completeness of cases and deaths compared with official national totals. Data on vaccination status of the children in this study was not available, however, during the study period, vaccines were only approved in the EU for use in children 12–17 years-old. As children are less likely to be symptomatic for COVID-19 than adults [11,12], reporting of cases may be biased towards those with severe disease. Although we recoded 640 cases with hospitalisation date before date of onset as not hospitalised and the TESSy reporting protocol defined a hospitalised case as one with severe COVID-19 requiring admission to hospital or ICU [13], it is possible that some remaining reported paediatric COVID-19 hospitalisations may have been for other causes. While this could overestimate the crude risk of hospitalisation, we are aware that hospitalisations were under-reported to TESSy in three of the countries in our study (50–76% complete for Germany, Ireland and Sweden) for which comparison against publicly available admission data was possible, which would have the opposite effect. Overall, our results are comparable to those reported among symptomatic cases in a recent systematic review, including studies from Europe and the US [10]. Reporting of comorbidities was less likely among cases with severe outcomes, making our effect estimates conservative for cases with a comorbidity. Low numbers of children with severe outcomes diminish the analytical power of the study, particularly for less common outcomes. This, together with incomplete reporting of comorbidities, prevented a detailed risk factor analysis.

## Conclusions

Paediatric hospital admissions for COVID-19 increased as overall transmission rates increased. The individual risks of a severe COVID-19 outcome were substantially elevated for those with a comorbidity compared with healthy children, but most children hospitalised in this study with data on comorbidities had no reported comorbidity. This demonstrates the additive impact of high levels of community transmission and can inform decision making around paediatric COVID-19 vaccinations. Preventive measures to reduce transmission and severe outcomes in children remain critical, as does the submission of timely, complete surveillance data to facilitate assessment of severity following the emergence of new variants. 
